# Correlates of Changes in Physical Activity and Sedentary Behaviors during the COVID-19 Lockdown in France: The NutriNet-Santé Cohort Study

**DOI:** 10.3390/ijerph191912370

**Published:** 2022-09-28

**Authors:** Hélène Charreire, Charlotte Verdot, Fabien Szabo de Edelenyi, Mélanie Deschasaux-Tanguy, Bernard Srour, Nathalie Druesne-Pecollo, Younes Esseddik, Benjamin Allès, Julia Baudry, Valérie Deschamps, Benoit Salanave, Pilar Galan, Serge Hercberg, Chantal Julia, Emmanuelle Kesse-Guyot, Sapris Study Group, Alice Bellicha, Mathilde Touvier, Jean-Michel Oppert

**Affiliations:** 1 Université Paris-Est Créteil, Lab’Urba, 94000 Créteil, France; 2 Sorbonne Paris Nord University, Institut National de la Santé et de la Recherche Médicale (INSERM), Institut National de Recherche pour l’Agriculture, l’Alimentation et l’Environnement (INRAE), Conservatoire National des Arts et Métiers (CNAM), Nutritional Epidemiology Research Team (EREN), Epidemiology and Statistics Research Center–University Paris Cité (CRESS), 93017 Bobigny, France; 3 Institute Universitaire de France (IUF), 75005 Paris, France; 4 Sorbonne Paris Nord University, Institut National de la Santé et de la Recherche Médicale (INSERM), Institut National de Recherche pour l’Agriculture, l’Alimentation et l’Environnement (INRAE), Conservatoire National des Arts et Métiers (CNAM), Nutritional Surveillance and Epidemiology Team (ESEN), French Public Health Agency Epidemiology and Statistics Research Center–University Paris Cité (CRESS), 93017 Bobigny, France; 5 Public Health Department, Avicenne Hospital, AP-HP, 93000 Bobigny, France; 6 Human Nutrition Research Center Ile-de-France (CRNH IdF), Pitié-Salpêtrière Hospital (AP-HP), Department of Nutrition, Sorbonne University, 75013 Paris, France

**Keywords:** longitudinal study, physical activity, sedentary behavior, COVID-19, lockdown, adults

## Abstract

Background: COVID-19 lockdowns represent natural experiments where limitations of movement impact on lifestyle behaviors. The aim of this paper was to assess how lockdowns have influenced physical activity and sedentary behaviors among French adults. Methods: 32,409 adults from the NutriNet-Santé study filled out questionnaires in April 2020 (the first 2 weeks after the start of lockdown) and in May 2020 (2 weeks before the lockdown ended). Participants were asked about changes in physical activity level and sitting time, types of physical activity performed, and main reasons for change. Results: For decreased physical activity, similar rates were found at the beginning and end of the lockdown (58 and 55%–56 and 53%, in women and men, respectively). For increased physical activity, the figures were lower (20 and 14%–23 and 18%, in women and men, respectively). The participants with a decreasing physical activity evolution were older and more likely to be living in urban areas. The main reasons for (i) decreased physical activity were limitations of movement and not liking indoor exercise, (ii) increased physical activity were to stay physically fit and healthy. Physical activity changes were inversely associated with reported depressive symptoms. Conclusions: Changes in physical activity and sedentary behaviors are heterogenous for both genders during the lockdown.

## 1. Introduction

To counter the overall spread of the COVID-19 infection and the associated burden on health care systems, many countries put periods of lockdown (stay-at-home and quarantine orders) in place during the year 2020 [1]. In France, the initial strict national lockdown measures took place from 17 March to 11 May 2020 and included the closure of all but the most essential public places, businesses and services, prohibition of going outdoors except for essential needs (e.g., food shopping, medical care, legal obligations) and permitting only 1 hour of recreational activity in a 1 km radius around residential address. Forests, parks, and beaches were also closed to the public. In addition, going out of home required a written certificate with valid reasons that had to be carried at all times. A majority of the working population was required to work from home or was placed on partial/technical unemployment. Schools were closed, and children stayed at home. Such drastic measures imposed at the national level resulted in an unprecedented disruption of daily life. It may be viewed as an unplanned natural experiment that may be used to understand the effects of an enforced limitation of movement on health behaviors such as physical activity and sedentary behaviors.

The benefits of physical activity on health are indisputable. Physical activity has a major preventative effect on the incidence of non-communicable diseases, particularly coronary heart disease, type 2 diabetes, several types of cancer (e.g., breast and colon cancers) and it is associated with improvements in mental health (e.g., depression) [2]. Therefore, changes in usual physical activity that could take place in the COVID-19 lockdown setting are likely to have non-negligible health consequences. Interestingly, although a reduction in overall physical activity would be expected in the COVID-19 lockdown context, it is noticeable that physical activity was found to actually increase in some studies or subgroups during lockdown [3,4,5,6]. In a recent systematic review based on 57 studies from 14 countries worldwide, a majority of studies (32 out of 57) reported a significant decrease in physical activity during lockdowns, but five of these studies observed a significant increase in physical activity, whereas six studies reported no change and fourteen noted both an increase and decrease in physical activity [7]. In France, a previous study of our group in a sample of more than 37,000 adult participants of the French NutriNet web-cohort, showed that, during this period, more than half (53%) of the participants reported decreased physical activity, whereas one fifth (19%) reported an increase [5]. Importantly, not only did the lockdown modify physical activity, but it also impacted sedentary behavior (mainly referring to sitting or screen time) [8].

In France, a COVIPREV survey based on 2000 adults observed that 47% of the participants reported decreased physical activity, while 61% of them increased daily sitting time during strict lockdown [4]. In this study, the authors showed that 49% of men and 53% of women did not meet physical activity recommendations (at least 30 minutes of physical activity/day) in such setting [4]. These rates are much higher than those found in the general French population before the pandemic (29% and 47% in men and women, respectively) [9]. The National Observatory of Physical Activity and Sedentary Behaviors (ONAPS) developed a specific lockdown-survey including 15,226 adults (18–64 years) and noted that 24.6% of adults increased their sitting time, and 41% increased their screen time during lockdown [10]. In another study, an online survey conducted among 4005 adults showed that more than 8 in 10 respondents reported decreased physical activity and more specifically decreased walking (60%) and exercising (45%) in parallel with increased screen watching (59%) [11]. It has to be underlined that in these previous studies, detailed information on types of physical activity performed and reasons for change in the lockdown setting was lacking

Therefore, the research question we asked was: In which way do strict lockdown measures influence physical activity and sedentary behaviors among adults? More detailed reports of changes in specific activities (e.g., walking, aerobic exercise, dancing, gardening) according to gender characteristics may improve our understanding of the different trends (decrease, increase) occurring in such settings. In addition, it is somewhat obvious that the initial reason why physical activity levels were altered during the COVID-19 pandemic is the social and spatial restrictions during the lockdown. However, investigation of specific reasons or motivations for participants to change their physical activity remains limited, especially in the French context. In previous studies in Canada, Belgium and Brazil, the lack of appropriate facilities/equipment/space [12,13,14], the lack of interest/motivation and the lack of time were reported as the main barriers to perform physical activity during lockdown.

Improved knowledge of the specific motivations for those doing more or less physical activity during lockdown may also deepen our understanding of individual barriers or levers to perform physical activity, which could be useful in other settings.

Therefore, the aims of this study were: (i) to explore physical activity and sedentary behaviors over the initial strict lockdown period that occurred in metropolitan France during March to May 2020 and reasons for changes as reported by participants, (ii) to investigate which type of physical activity had been performed during lockdown, and (iii) to identify correlates of change in physical activity across the strict lockdown period. Such knowledge will help designing targeted public health interventions beyond the event of further lockdowns.

## 2. Materials and Methods

### 2.1. Study Population: The NutriNet-Santé Cohort

Individual data regarding physical activity, sedentary behaviors and other individual covariates were collected from participants of the NutriNet-Santé study, an ongoing web-based cohort launched in France in May 2009, which focuses on the study of relationships between nutrition and health. This cohort has been previously described in detail [15]. Volunteers aged 18 years or older living in France and having access to the Internet fill in self-administered web-based questionnaires at baseline as well as at regular time points during follow-up using a dedicated website. Moreover, the participants also completed a set of questionnaires assessing demographic and socioeconomic characteristics [16]. The study uses a secure and flexible online platform for recruitment and data collection (www.etude-nutrinet-sante.fr). It allows for the rapid implementation of ad hoc research protocols. The study was initiated and is managed by the Nutritional Epidemiology Research Team (EREN), Epidemiology and Statistics Research Center–University Paris Cité (CRESS), Bobigny, France. All NutriNet-Santé questionnaires are available online (in French): https://info.etude-nutrinet-sante.fr/siteinfo/article/52 (accessed on 24 September 2022).

### 2.2. Data Collection during the COVID-19 Lockdown

#### 2.2.1. Physical Activity and Sedentary Behaviors

In April and in May 2020, a set of ad hoc questionnaires was sent to the NutriNet-Santé participants to collect extensive data on health status and health behaviors, including physical activity at the beginning (T1) and during the lockdown (T2). Similar questions were asked at the two time points (T1 and T2). During the first 15 days of lockdown (T1), i.e., early April 2020, participants were asked about whether they had increased, decreased, or not modified their overall physical activity compared to their habitual level of physical activity before the lockdown. Detailed information was collected on different types of physical activity performed, asking if the activity under question had been performed earlier (and, if so, whether its duration had increased or decreased since the beginning of the lockdown) or if the activity had been started at the beginning of the lockdown. Activities surveyed included: brisk walking, walking an animal, jogging, cycling (outdoors and indoors), treadmill walking/running, rowing, aerobics, dance, strength training, yoga, stretching, active play with children, household chores (cleaning), gardening and craft activities, and other activity (write in the space provided). At both time points (T1 and T2), participants were also asked to report the main reason for changes in overall physical activity.

#### 2.2.2. Covariates

Another questionnaire assessing social and demographic participants’ exposure to SARS-CoV-2, COVID-19 infection was sent in April 2020 as part of a nation-wide multi-cohort project including the NutriNet-Santé participants (Health, practices, relationships, and social inequalities in the general population during the COVID-19 crisis, SAPRIS, [17]). This questionnaire was used to derive demographic information (professional activity change, residing with a partner (yes/no), presence of children or grandchildren aged < 18 years at home), presence of chronic disease (yes/no), mental health information (presence of depressive symptoms using the Patient Health Questionnaire (PHQ)-9 scale) [18]; anxiety (using the General Anxiety Disorder (GAD)-7 scale) [19] and self-reported body weight measures during the lockdown. Body mass index (BMI) was computed as weight [kilograms]/height² [meters²]. Body weight status was categorized as overweight (BMI ≥ 25 kg/m²), and non-overweight (BMI < 25 kg/m²).

### 2.3. Statistical Analyses

A total of 32,409 participants completed the specific physical activity questionnaire sent during the COVID-19 lockdown at both time points (early April–T1 and early May 2020–T2). Among these, 23,558 participants had also completed the SAPRIS questionnaire (in April 2020) with covariables of interest for the present analyses. Data were summarized using numbers and percentages for categorical variables and mean values and standard deviations (or median values and interquartile ranges) for continuous variables. Data were categorized according to changes reported in overall physical activity at the beginning (T1) and later during lockdown (T2). Subjects were grouped into four categories according to changes in physical activity: (1) “Increased physical activity”: those who reported an increase at both time points or who reported an increase and then stability, (2) “Decreased physical activity”: those reporting a decrease at both time points or who reported a decrease and then stability, (3) “Stable”: those who reported no change at both time points, and (4) “Others”: those who had any other combination of increased/decreased/stable physical activity, for example who reported an increase at the first time point and then a decrease. All analyses were performed by gender. ANCOVA was used to compare quantitative variables, and Chi-square tests were used to compare categorical variables between the four types of physical activity change.

Multivariable logistic regressions were performed, by gender, to assess the relationship between each physical activity category of change and characteristics of the participants (category X vs. all others categories combined). The characteristics of the participants (detailed in Table 1) included: age, weight status, current smoking status, educational level, household monthly income, professional activity change during the lockdown, being essential providers or workers during lockdown, residing with a partner during the lockdown, presence of children and/or grandchildren aged < 18 years at home during the lockdown, habitual residential area during the lockdown, depressive symptoms during the lockdown (PHQ-9 score), anxiety during the lockdown (GAD-7 score), self-reported chronic disease, behavior changes and perceived snacking change during the lockdown. All tests were two-sided and *p* < 0.05 was considered statistically significant. Analyses were computed using SAS 9.4 (SAS Institute Inc., USA).

## 3. Results

The characteristics of the study population (at T1) according to gender (*n* = 17,364 women (73.7%) and *n* = 6194 men (26.3%)) are presented in Table 1.

### 3.1. Perceived Changes in Physical Activity and Sedentary Behavior

A majority of women (58%) and men (55.1%) reported that they had decreased their physical activity at T1 (Table 2). 

Similar results were observed at T2 with 56.4% and 52.6% of women and men reporting decreased physical activity, respectively. In contrast, 19.8% of women and 14% of men reported an increase in physical activity at T1, and 23.2% of women and 18.3% of men an increase in physical activity at T2 (*p* < 0.001 for comparison between genders). In parallel, 66.3% of women and 60.9% of men reported an increase in sitting time at T1, and 49.8% of women and 53.3% of men reported no change in sitting time at T2 (*p* < 0.001 for comparison between genders).

### 3.2. Main Reasons for Change in Physical Activity

**Figure 1:**
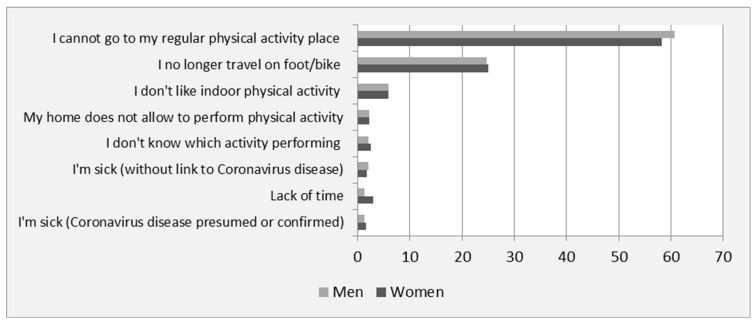
Main reasons (%) reported by women (*n* = 10,077) and men (*n* = 3413) to explain decreased physical activity at the beginning of the lockdown (T1).

The main reasons for decreased physical activity (shown in Figure 1) at T1 were as follows: difficulty to access regular places to perform physical activity (39.4% of women and 45% of men); lifestyle changes inherent to the lockdown situation such as stopping outdoor walking and cycling (18.2% and 19% of women and men, respectively) and preferences (do not like indoor physical activity for 16.6% of women and 15.4% of men). The hierarchy of reasons was the same at T2 for both genders (data not shown).

**Figure 2:**
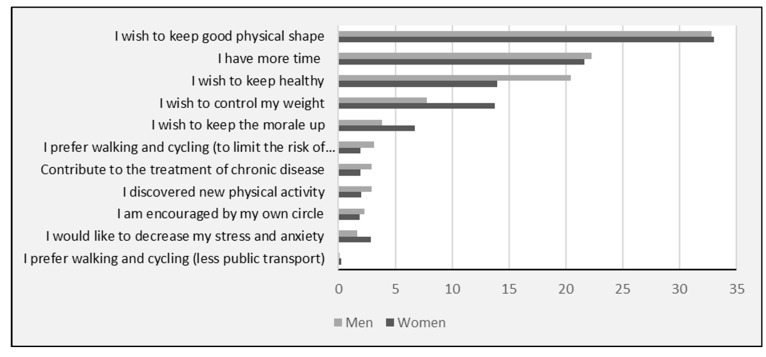
Main reasons (in %) reported by women (*n* = 3430) and men (*n* = 866) to explain increased physical activity (T1).

In contrast, the main reasons for increased physical activity at T1 (Figure 2) were related to voluntary changes in behavior in order to remain physically fit, to stay healthy, to better control body weight, or to improve mood. Subjects also reported having more time and the possibility to discover new types of physical activity. At T2, the main reason was still “to remain physically fit”, but the reason “I have more time” was downgraded to the 6th position behind reasons related to voluntary behavior changes (staying healthy, better controlling body weight, and improving mood) (data not shown).

### 3.3. Changes in Different Types of Physical Activity

**Figure 3:**
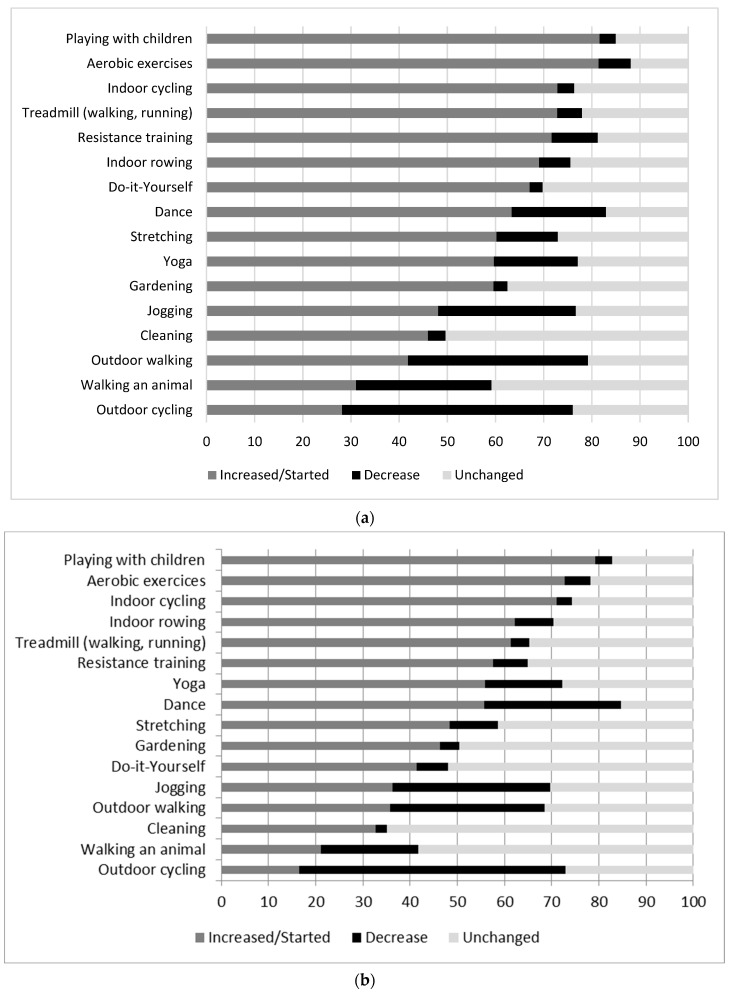
(**a**). Changes (increase, decrease or no change, %) for each type of physical activity among women at T1. (**b**). Changes (increase, decrease, no change, %) for each type of physical activity among men at T1.

Figure 3a,b show the proportion of women and men, respectively, reporting a decrease, an increase, or no change in specific types of physical activity. For both women and men, activities most frequently reported as increased were playing with children, aerobic exercises, and indoor cycling. Activities most frequently reported as decreased were outdoor cycling, walking, and jogging. Descriptive statistics on characteristics of participants according to categories of change in physical activity are presented in Additional File S1 (Tables S1 and S2 for women and men, respectively).

### 3.4. Characteristics of Participants According to Physical Activity Changes

Results of analyses using multivariable logistic regression modelling to assess relationships between characteristics of subjects and categories of physical activity change are presented in Table 3a,b, for women and men, respectively. For both genders, participants in the “decrease physical activity” category were more likely (than the other categories) to report increased sedentary time (OR = 2.5 [CI 2.3–2.7]; OR = 3.1 [CI 2.7–3.5] for women and men, respectively). This category was associated with older age and living in urban areas. Women in this group were more likely to report having children/grandchildren at home, no change in professional activity during the lockdown (e.g., change is working from home/new job) and more “moderately severe” to “severe” depressive symptoms compare to women with mild depressive symptoms and the other categories.

Participants in the “Increase physical activity” category (men and women) were more likely to report decreased sedentary time (OR = 2.2 [CI 1.9–2.6]; OR = 2.6 [1.6–2.9] for women and men, respectively) compared to the other categories. This category was associated with younger age, not having a partner and children/grandchildren at home (only for women) and living in a rural area. Women reported having significantly more changes in professional activity (than women with no professional activity and in comparison, to other categories) and men were characterized by being more frequently essential providers and workers during the lockdown. Among women, the increased physical activity category was also associated with less “moderately severe” to “severe” depressive symptoms compared to women with mild depressive symptoms and the other categories.

For both genders, the participants in the “stable physical activity” category were more likely to report no change in sedentary time during the lockdown than the other categories. Women in this category, were more likely to report minimal-to-mild depressive symptoms, to be categorized as with overweight, to be current smokers, to have a low level of monthly income, to be living in a rural area, and to report no change in professional activity during the lockdown. Men in this category were more likely to be categorized as with overweight, to be current smokers, to have a low level of education, to have no partner during the lockdown and to be living in a rural area.

Finally, participants in the “other” category were more likely to report perceived favorable changes for men except for snacking (more snacking during the day during lockdown). In addition, these participants reported heterogenous sedentary time changes (increase or decrease). This category was associated with younger age (among both genders) and a higher likelihood for living in urban areas (for women). Among women, this profile was also associated with mild depressive symptoms and the absence of smoking.

## 4. Discussion

Our study, based on 23,558 adults, provides a detailed overview of physical activity changes at the beginning and during the COVID-19 lockdown in France and shows the heterogeneity of changes in self-reported physical activity behavior for both genders. While a majority of participants reported a decrease in physical activity and nearly two thirds of subjects reported an increase in sedentary time at the beginning of the lockdown, we also observed, in both genders, that some participants reported an increase or stability in their physical activity levels. These results are in line with some previous studies, which observed contrasted evolutions in physical activity habits in the lockdown context [20,21,22,23,24,25]. For example, in the UK, Bue et al. (2021) identified six specific weekly trajectories of physical activity behavior among a sample of 35,915 adults during (the initial strict) lockdown and following easing of restrictions (24th March–23rd August 2020) [20]. Three of these weekly trajectories (62.4% of the sample) were static (with little change observed), two were characterized by decreased physical activity over time (28.6% of the sample) and one trajectory was characterized by an overall increase in physical activity over time (9% of the sample).

In our study, those with a decreasing physical activity evolution during the lockdown were older and more likely to be living in urban areas. The higher probability for older participants to decrease physical activity may be explained by the higher perceived susceptibility to COVID-19 infection, which may more strongly limit collective and/or outdoor physical activity. Along the same lines, Ding et al. (2021) observed in 815 Chinese adults residing in Shanghai that those who were older recovered step counts at a slower pace than their younger counterparts after the lockdown [25]. In contrast, in the UK, adults who experienced decreasing levels of physical activity during lockdown were younger in a declarative study [20] and in a study based on users of smartphone-tracked activity [26]. As hypothesized, limitation of daily active commuting may have a stronger influence in physical activity in younger age groups.

In the current study, participants living in urban areas (vs. rural) had higher odds of decreasing physical activity evolution throughout the lockdown. This result may be partially explained by the fact that rural areas provide environments that make it easier than urban areas to get out (detached home with garden or yards, large natural environment) or to perform home exercises (home space) in the lockdown context. In contrast, Beck et al. (2021) found that urban area participants seemed to perceive more barriers such as size or layout of indoor space than rural areas participants [27]. Altogether, individual socio-demographic and living environment characteristics have complex and probably context-specific relationships with physical activity according to lockdown characteristics (duration, level of daily/professional restrictions) and perception of risk about COVID-19.

Our study also explored the main motives for self-reported changes in physical activity (decline or increase). Unsurprisingly, in our sample, the difficulty to access regular places to perform physical activity, the impossibility by law of walking and cycling outside (especially trips for commuting and shopping, except for essential goods) and lack of interest in physical activity ranked first as reasons to explain decreased physical activity for both genders. In Brazil, Farah et al. (2021) observed that “Laziness, fatigue”, “Lack of motivation”, “Lack of appropriate facilities/equipment/space” and “Lack of time” were the most prevalent barriers reported by Brazilian adults during social isolation measures due to the COVID-19 pandemic [14]. In contrast to results in other countries [12,14,28], lack of time is rarely reported in our study (2.9% women and 1.3% men). It could possibly be because of age (35% of participants were 65 y or more—hence likely retired) and/or because of absence of professional activity during this period.

Regarding motivations for those doing more physical activity during lockdown, the main reasons were related to maintenance of physical fitness and then overall health in men or controlling weight in women. Garcia-Tascon et al. observed similar findings in Spanish adults for whom the main motivations to perform physical activity during lockdown were being fit, having fun (for men) and staying healthy and relaxing (for women) [28]. Interestingly, although outdoor physical activity was restricted to no more than one hour per day in a 1 km radius around the home, the most frequent activity reported as started during the lockdown was outdoor walking for men. An explanation could be that in countries such as France, UK or Greece [3] outdoor physical activity (such as walking—especially with dogs—and jogging) was considered an “essential activity” during the lockdown.

Our study confirms the heterogeneity of changes in physical activity and sedentary behaviors for both genders in the lockdown setting.

Regarding gender stratification, in previous studies, although men tended to be more active than women during the COVID-19 pandemic, a lower decrease in the quantity of physical activity was observed for women compared to men [28,29]. Importantly, the negative impact of the lockdown and social isolation on mental health was observed specifically for women [30,31,32,33,34]. In our sample, women who have a decreasing physical activity evolution have significantly higher odds of depressive symptoms compared with other types of change, even after accounting for potential confounding factors. These results are consistent with previous studies among Brazilian [35], Italian [36] and US adults [22] for whom reported decreases in physical activity during lockdown were associated with poorer mental health indicators, especially in women. Interestingly, our study also shows that individual characteristics such as living with children at home or to be considered as an essential provider or worker during lockdown do not have the same relation with the change of physical activity behavior in women and men. The more frequent type of physical activity started at the beginning of the lockdown was also different by gender. Thus, the data point to a non-uniform impact of the lockdown on physical activity behavior in women and men, possibly leading to the development of gender-specific interventions in such settings [37].

## 5. Strengths and Limitations

Our study has several important strengths, including the large sample size allowing analyses stratified by gender, very detailed information on changes in specific types of physical activity as well as data on the reasons for subjects to increase or decrease physical activity behaviors during that unique time period. Some limitations should be mentioned. First, our study relies on self-reported data, and most questions used in the current analyses focused on the perceived changes during the lockdown, which may be prone to social desirability bias. The duration of reported physical activities was not assessed. Second, we did not consider information about physical activity and sedentary behaviors during the post-lockdown period that would help assess whether behaviors were (or were not) maintained over time. Finally, the generalization of our findings may be limited in the sense that relations may be context-specific for each country (also depending on measures imposed by governments during the pandemic [38]) and our sample included more women than in the general population, as well as more individuals with higher education.

## 6. Conclusions

In conclusion, investigation of both the reasons why women and men are showing changes in physical activity and type of physical activity performed provide elements to better understand both unhealthy as well as healthy behaviors during periods of restriction of movement. The different patterns of evolution we observed underline the need to adapt clear messages to different populations according to socio-demographic characteristics and residential context (urban level) when promoting physical activity in constrained settings. As outdoor physical activity such as active mobility (walking and cycling) and jogging were the activities that were most impacted by restrictions during the lockdown, public authorities should be aware of the need to keep outdoor places for physical activity (such as parks) open, especially in urban areas. For those who started activities during the lockdown, the challenge will be to maintain these activities over time, leading to the importance of post-lockdown studies. It will be interesting to further investigate the barriers to physical activity during (i) different levels (and time) of restriction in pandemic context at national and international level and (ii) during the period after the lockdown. Finally, data on relationships with depressive symptoms highlight the value of physical activity as an important marker of overall and mental health, especially in women, to be included in surveillance efforts and public health policies at national and international level.

## Figures and Tables

**Table 1:** Characteristics of the study population according to gender.

	Women (*n* = 17,364, 73.7%)		Men (*n* = 6194, 26.3%)	
	*n*	%	*n*	%
Age (years)				
18–35	1661	9.6	274	4.4
36–50	4063	23.4	921	14.9
51–65	6506	37.5	1849	29.9
>65	5134	29.6	3150	50.9
Educational level				
<High-school degree	2479	14.3	1346	21.7
High-school degree	2268	13.1	684	11.0
Undergraduate degree	5746	33.1	1608	26.0
Graduate degree	6701	38.6	2504	40.4
Unknown	170	1.0	52	0.8
Monthly income (€ per household)				
<1430	1059	6.1	211	3.4
1430–2700	4047	23.3	1173	18.9
2700–4800	6255	36.0	2745	44.3
≥4800	3165	18.2	1471	23.7
Unknown	498	2.9	*73*	1.2
Did not wish to answer	2340	13.5	521	8.4
Reside with partner during the lockdown				
Yes	11,908	68.6	4882	78.8
No	1431	8.2	238	3.8
No partner	4025	23.2	1074	17.3
Children or grandchildren at home during the lockdown [yes]	3744	21.6	917	14.8
Professional activity changes during lockdown				
No professional activity (unemployed, retired, homemaker, maternity leave) or no professional activity during the last seven days (short-term leave).	10,764	62.0	4422	71.4
No change	1807	10.4	442	7.1
Yes, change (working from home, new job)	4410	25.4	1262	20.4
Other	383	2.2	68	1.1
Essential providers or workers during lockdown				
Yes	2565	14.8	517	8.3
No	14,040	80.9	5498	88.8
Don’t know	759	4.4	179	2.9
Residential area during the lockdown (number of inhabitants)				
Urban area >100,000	3449	19.9	1176	19.0
Urban area 20,000 to 100,000	3969	22.9	1330	21.5
Urban area <20,000	4011	23.1	1527	24.7
Rural area	5935	34.2	2161	34.9
Smoking status				
Never smoker	16,210	93.4	5830	94.1
Occasional smoker	289	1.7	103	1.7
Current smoker	865	5.0	261	4.2
Current weight status				
Overweight	5424	31.2	2806	45.3
Non-overweight	11,840	68.2	3359	54.2
Unknown	100	0.6	*29*	0.5
Chronic disease				
Yes	6152	35.4	2779	44.9
No	11,078	63.8	3366	54.3
Don’t know	134	0.8	49	0.8
PHQ-9 (depressive symptoms)				
Mild	3872	22.3	774	12.5
Minimal	11,884	68.4	5179	83.6
Moderate	1064	6.1	156	2.5
Moderately severe to severe	544	3.1	85	1.4
GAD-7 (anxiety disorders)				
Mild	3502	20.2	697	11.3
Minimal	12,383	71.3	5284	85.3
Moderate	949	5.5	141	2.3
Severe	530	3.1	72	1.2

**Table 2:** Physical activity and sitting time changes during the lockdown reported by participants according to gender.

	T1				T2			
	Women		Men		Women		Men	
	(*n* = 17,364)		(*n* = 6194)		(*n* = 17,364)		(*n* = 6194)	
	*n*	%	*n*	%	*n*	%	*n*	%
**Physical activity**								
Increased	3436	19.8	867	14.0	4036	23.2	1134	18.3
Decreased	10,077	58.0	3413	55.1	9794	56.4	3255	52.6
Unchanged	3851	22.2	1914	30.9	3326	19.2	1770	28.6
Don’t know					208	1.2	35	0.6
**Sitting time**								
Increased	11,485	66.1	3755	60.6	6531	37.6	2381	38.4
Decreased	854	4.9	201	3.2	1248	7.2	275	4.4
Unchanged	4384	25.2	2069	33.4	8654	49.8	3299	53.3
Don’t know	590	3.4	143	2.3	842	4.8	210	3.4
Missing	51	0.3	26	0.4	89	0.5	29	0.5

**Table 3:** **a**. Relationships between characteristics of women and categories of change in overall physical activity during the lockdown (T1 vs. T2). **b**. Relationships between characteristics of men and categories of change in overall physical activity during the lockdown (T1 vs. T2).

(a)												
	Decrease Category (*n* = 9090, 53.3%)			Increase Category (*n* = 3519, 20.3%)			Stable Category (*n* = 2140, 15.3%)			Other Category (*n* = 2651, 12.1%)		
	OR	95% CI		OR	95% CI		OR	95% CI		OR	95% CI	
Age (years)												
18–35	0.91	0.80	1.03	1.07	0.93	1.24	1.12	0.94	1.35	0.96	0.82	1.14
36–50	1.00	Ref		1.00	Ref		1.00	Ref		1.00	Ref	
51–65	1.48	1.33	1.63	0.72	0.64	0.81	0.94	0.81	1.09	0.79	0.69	0.92
>65	2.08	1.85	2.35	0.47	0.40	0.54	0.97	0.82	1.15	0.60	0.50	0.72
Educational level												
<High-school degree	1.08	0.96	1.22	0.89	0.76	1.04	1.03	0.88	1.20	0.87	0.71	1.07
High-school degree	1.00	Ref		1.00	Ref		1.00	Ref		1.00	Ref	
Undergraduate degree	0.95	0.85	1.05	1.08	0.95	1.23	0.96	0.83	1.10	1.09	0.93	1.28
Graduate degree	1.00	0.90	1.11	0.97	0.85	1.11	0.87	0.75	1.01	1.24	1.05	1.46
Unknown	0.96	0.67	1.36	1.00	0.65	1.55	0.87	0.53	1.41	1.30	0.77	2.21
Monthly income (€ per household)												
<1430	1.00	Ref		1.00	Ref		1.00	Ref		1.00	Ref	
1430 to 2700	1.16	1.00	1.34	0.98	0.81	1.17	0.78	0.64	0.94	0.97	0.78	1.22
2700 to 4800	1.10	0.95	1.27	1.11	0.92	1.33	0.65	0.53	0.80	1.12	0.89	1.40
≥4800	1.00	0.85	1.18	1.11	0.90	1.35	0.67	0.54	0.84	1.31	1.03	1.68
Unknown	0.88	0.70	1.12	1.18	0.89	1.56	0.79	0.57	1.08	1.31	0.93	1.83
Did not wish to answer	1.11	0.94	1.30	0.98	0.80	1.20	0.83	0.67	1.03	0.97	0.76	1.25
Reside with partner during the lockdown												
Yes	1.10	0.97	1.24	0.80	0.69	0.92	1.13	0.94	1.35	1.00	0.84	1.19
No	1.00	Ref		1.00	Ref		1.00	Ref		1.00	Ref	
No partner	1.12	0.98	1.28	0.84	0.72	0.98	0.94	0.77	1.15	1.07	0.88	1.30
Children or grandchildren at home during the lockdown												
Yes	1.00	Ref		1.00	Ref		1.00	Ref		1.00	Ref	
No	0.83	0.75	0.91	1.25	1.12	1.41	0.93	0.81	1.08	1.11	0.97	1.28
Professional activity changes during the lockdown												
No professional activity (unemployed, retired, homemaker, maternity leave) or no professional activity during the last seven days (short-term leave).	1.00	Ref		1.00	Ref		1.00	Ref		1.00	Ref	
No change	1.17	1.01	1.36	0.74	0.62	0.89	1.25	1.03	1.54	0.75	0.60	0.93
Yes, change (working from home, new job)	0.91	0.82	1.00	1.15	1.03	1.29	0.89	0.76	1.03	1.04	0.90	1.19
Other	0.93	0.74	1.17	0.94	0.71	1.24	1.29	0.95	1.76	0.96	0.69	1.34
Essential providers or workers during lockdown												
Yes	1.11	0.98	1.25	0.80	0.69	0.92	1.16	0.97	1.38	0.96	0.81	1.14
No	1.00	Ref		1.00	Ref		1.00	Ref		1.00	Ref	
Don’t know	1.07	0.91	1.26	0.94	0.78	1.14	0.92	0.70	1.20	1.01	0.81	1.27
Residential area during the lockdown (number of inhabitants)												
Urban area >100.000	1.30	1.19	1.43	0.77	0.69	0.87	0.67	0.59	0.77	1.25	1.10	1.43
Urban area ≥20.000 to 100.000	1.32	1.21	1.44	0.83	0.75	0.93	0.68	0.60	0.77	1.12	0.99	1.28
Urban area <20.000	1.21	1.12	1.32	0.86	0.78	0.96	0.80	0.71	0.90	1.12	0.99	1.28
Rural area	1.00	Ref		1.00	Ref		1.00	Ref		1.00	Ref	
Smoking status												
Never smoker	1.00	Ref		1.00	Ref		1.00	Ref		1.00	Ref	
Occasionally smoker	0.98	0.77	1.25	1.14	0.86	1.51	1.29	0.92	1.80	0.62	0.42	0.93
Current smoker	1.10	0.95	1.27	0.83	0.69	0.99	1.54	1.28	1.87	0.61	0.48	0.78
Current weight status												
Overweight	0.99	0.92	1.06	0.93	0.85	1.02	1.16	1.06	1.28	0.95	0.86	1.06
Non-overweight	1.00	Ref		1.00	Ref		1.00	Ref		1.00	Ref	
Unknown	1.08	0.69	1.70	1.10	0.65	1.87	0.85	0.43	1.68	0.79	0.40	1.57
Chronic disease												
Yes	1.02	0.95	1.09	0.93	0.86	1.02	1.05	0.96	1.16	1.01	0.92	1.12
No	1.00	Ref		1.00	Ref		1.00	Ref		1.00	Ref	
Don’t know	0.83	0.58	1.19	1.43	0.95	2.16	1.39	0.87	2.22	0.51	0.26	1.01
PHQ-9 (depressive symptoms)												
Mild	1.08	0.99	1.17	0.86	0.77	0.95	0.86	0.75	0.97	1.22	1.08	1.38
Minimal	1.00	Ref		1.00	Ref		1.00	Ref		1.00	Ref	
Moderate	1.11	0.96	1.30	0.82	0.68	0.99	0.91	0.72	1.15	1.14	0.93	1.41
Moderately severe to severe	1.43	1.14	1.79	0.65	0.48	0.88	0.68	0.47	0.96	1.16	0.85	1.58
GAD-7 (anxiety disorders)												
Mild	1.00	Ref		1.00	Ref		1.00	Ref		1.00	Ref	
Minimal	0.97	0.89	1.06	1.01	0.91	1.12	1.02	0.90	1.16	1.02	0.90	1.15
Moderate	0.99	0.85	1.16	0.99	0.81	1.20	1.06	0.84	1.34	0.98	0.78	1.22
Severe	1.03	0.82	1.28	0.71	0.52	0.97	1.37	0.99	1.88	1.06	0.78	1.43
Sedentary time change												
Decrease	0.75	0.64	0.89	2.23	1.90	2.62	0.26	0.24	0.29	1.47	1.16	1.86
Increase	2.47	2.29	2.67	0.65	0.59	0.71	0.44	0.35	0.54	1.77	1.55	2.01
No change	1.00	Ref		1.00	Ref		1.00	Ref		1.00	Ref	
Don’t know	1.03	0.86	1.24	1.52	1.25	1.84	0.52	0.42	0.65	1.52	1.15	2.01
Missing data	2.87	1.61	5.13	0.69	0.33	1.44	0.43	0.20	0.94	0.48	0.12	1.99
**(b)**												
	**Decrease Category** **(*n* = 3336, 53.9%)**			**Increase Category** **(*n* = 983, 15.9%)**			**Stable Category** **(*n* = 1395, 22.5%)**			**Other Category** **(*n* = 480, 7.7%)**		
	**OR**	**95% CI**		**OR**	**95% CI**		**OR**	**95% CI**		**OR**	**95% CI**	
Age (years)												
18–35	0.72	0.53	0.97	1.09	0.76	1.57	1.36	0.88	2.1	1.19	0.8	1.75
36–50	1	Ref		1	Ref		1	Ref		1	Ref	
51–65	1.21	1	1.48	0.9	0.71	1.15	1.23	0.93	1.63	0.64	0.48	0.87
>65	1.49	1.19	1.87	0.61	0.46	0.82	1.35	0.99	1.85	0.5	0.34	0.72
Educational level												
<High-school degree	0.94	0.77	1.14	0.94	0.71	1.24	1.43	1.13	1.81	0.45	0.3	0.68
High-school degree	1	Ref		1	Ref		1	Ref		1	Ref	
Undergraduate degree	0.98	0.81	1.19	1.04	0.8	1.35	1.13	0.89	1.44	0.77	0.56	1.08
Graduate degree	0.92	0.76	1.11	1.05	0.81	1.35	1.2	0.94	1.52	0.86	0.63	1.19
Unknown	1.16	0.6	2.24	0.56	0.2	1.59	1.54	0.72	3.32	0.78	0.23	2.64
Monthly income (€ per household)												
<1430	1	Ref		1	Ref		1	Ref		1	Ref	
1430 to 2700	1.01	0.73	1.38	1.06	0.68	1.65	0.79	0.54	1.16	1.38	0.75	2.54
2700 to 4800	1.06	0.77	1.45	1.03	0.67	1.6	0.73	0.5	1.07	1.58	0.86	2.88
>=4800	0.89	0.64	1.25	1.49	0.94	2.35	0.63	0.42	0.95	1.57	0.84	2.95
Unknown	0.91	0.5	1.66	1.88	0.9	3.9	0.75	0.36	1.58	0.58	0.16	2.1
Did not wish to answer	1.09	0.76	1.55	0.88	0.54	1.45	0.8	0.52	1.22	1.5	0.76	2.96
Reside with partner during the lockdown												
Yes	0.9	0.67	1.2	0.97	0.67	1.41	1.28	0.84	1.95	1	0.63	1.56
No	1	Ref		1	Ref		1	Ref		1	Ref	
No partner	0.83	0.61	1.13	0.87	0.59	1.3	1.6	1.03	2.47	0.95	0.59	1.54
Children or grandchildren at home during the lockdown												
Yes	1	Ref		1	Ref		Ref			1	Ref	
No	0.92	0.77	1.11	0.98	0.78	1.24	1.07	0.83	1.38	1.12	0.84	1.51
Professional activity changes during the lockdown												
No professional activity (unemployed, retired, homemaker, maternity leave) or no professional activity during the last seven days (short-term leave).	1	Ref		1	Ref		Ref			1	Ref	
No change	1.1	0.83	1.45	0.64	0.44	0.93	1.15	0.81	1.65	1.09	0.7	1.71
Yes. change (working from home. new job)	1.08	0.89	1.31	0.94	0.74	1.19	0.87	0.66	1.14	0.95	0.71	1.29
Other	0.7	0.4	1.2	1.2	0.63	2.3	1.21	0.61	2.4	1.29	0.56	2.95
Essential providers or workers during lockdown												
Yes	1.02	0.8	1.3	2.65	1.92	3.64	1.25	0.9	1.72	0.87	0.59	1.28
No	1	Ref		1	Ref		Ref			1	Ref	
Don’t know	1.05	0.75	1.47	0.86	0.55	1.33	1.68	1.08	2.62	0.62	0.34	1.11
Residential area during the lockdown (number of inhabitants)												
Urban area >100.000	1.58	1.34	1.85	0.84	0.68	1.03	0.55	0.44	0.68	1.21	0.91	1.61
Urban area >=20.000 to 100.000	1.55	1.33	1.79	0.74	0.6	0.91	0.64	0.53	0.77	1.22	0.92	1.61
Urban area <20.000	1.31	1.14	1.51	0.94	0.78	1.13	0.68	0.58	0.8	1.25	0.96	1.64
Rural area	1	Ref		1	Ref		Ref			1	Ref	
Smoking status												
Never smoker	1	Ref		1	Ref		1	Ref		1	Ref	
Occasionally smoker	1.16	0.76	1.76	0.9	0.53	1.53	1.03	0.61	1.75	0.76	0.36	1.6
Current smoker	0.84	0.65	1.1	1.01	0.72	1.43	1.46	1.07	1.99	0.68	0.4	1.16
Current weight status												
Normal	1	Ref		1	Ref		1	Ref		1	Ref	
Overweight	0.91	0.81	1.01	1.07	0.92	1.25	1.18	1.03	1.35	0.91	0.38	2.2
Unknown	0.53	0.22	1.29	1.38	0.47	4.06	1.32	0.48	3.64	2.13	0.57	7.99
Chronic disease												
Yes	0.98	0.87	1.09	1.1	0.94	1.28	1.06	0.93	1.22	0.81	0.66	1.01
No	1	Ref		1	Ref		1	Ref		1	Ref	
Don’t know	0.84	0.46	1.54	1.65	0.78	3.47	0.85	0.39	1.82	1	0.34	2.88
PHQ-9 (depressive symptoms)												
Mild	0.95	0.79	1.14	0.89	0.7	1.14	1.02	0.81	1.3	1.27	0.95	1.69
Minimal	1	Ref		1	Ref		1	Ref		1	Ref	
Moderate	0.93	0.63	1.36	1.06	0.64	1.76	0.65	0.35	1.21	1.6	0.93	2.74
Moderately severe to severe	1.2	0.69	2.1	0.7	0.31	1.59	1	0.47	2.15	0.91	0.38	2.2
GAD-7 (anxiety disorders)												
Mild	1	Ref		1	Ref		Ref			1	Ref	
Minimal	0.88	0.73	1.06	1.02	0.8	1.31	1.18	0.92	1.51	1.05	0.77	1.43
Moderate	0.81	0.54	1.21	1.42	0.86	2.35	0.98	0.56	1.73	1.13	0.6	2.11
Severe	1.06	0.58	1.93	0.44	0.16	1.24	1.74	0.81	3.75	1.08	0.43	2.74
Sedentary time change												
Decrease	0.72	0.52	1.01	2.65	1.92	3.64	0.42	0.28	0.62	1.98	1.18	3.32
Increase	3.1	2.75	3.49	0.63	0.53	0.74	0.24	0.21	0.28	2.04	1.57	2.66
No change	1	Ref		1	Ref		Ref			1	Ref	
Don't know	1.86	1.31	2.64	0.78	0.48	1.28	0.56	0.38	0.82	1.38	0.64	2.99
Missing data	1.04	0.46	2.34	0.51	0.15	1.77	0.87	0.37	2.02	/	/	/

## Data Availability

Researchers from public institutions can submit a collaboration request including their institution and a brief description of the project to collaboration@etude-nutrinet-sante.fr. All requests will be reviewed by the steering committee of the NutriNet-Santé study. A financial contribution may be requested. If the collaboration is accepted, a data access agreement will be necessary and appropriate authorizations from the competent administrative authorities may be needed. In accordance with existing regulations, no personal data will be accessible.

## Supplementary Materials

The following supporting information can be downloaded at: https://www.mdpi.com/article/10.3390/ijerph191912370/s1, Table S1. Characteristics of the women participants according to changes in physical activity during the lockdown. Table S2. Characteristics of the men participants according to changes in physical activity during the lockdown.

## Abbreviations

ANCOVA	ANalysis of COVAriance
BMI	Body Mass Index
CNIL	Commission Nationale de l’Informatique et des Libertés
GAD-7	Generalized Anxiety Disorder–7 scale
IPAQ	International Physical Activity Questionnaire
COVID-19	Coronavirus disease 2019
PA	Physical Activity
PHQ-9	Patient Health Questionnaire–9 scale
SARS-CoV-2	Severe Acute Respiratory Syndrome CoronaVirus 2
