# Supplementary material for: Correlates of Changes in Physical Activity and Sedentary Behaviors during the COVID-19 Lockdown in France: The NutriNet-Santé Cohort Study
Source: Int J Environ Res Public Health. 2022 Sep 28;19(19):12370. doi: 10.3390/ijerph191912370 (PMC9564572; doi:10.3390/ijerph191912370)
Supplement: Supplementary file 1 [file ijerph-19-12370-s001.zip › ijerph-1855140-supplementary.pdf]

Additional file S1

Table S1. Characteristics of the women participants according to changes in physical activity during the lockdown

	WOMEN (n = 17364)										
	Total	DECREASE		INCREASE		STABLE		OTHERS			P Value
		9090	(52.3)	3519	(20.3)	2651	(15.3)	2104	(12.1)		
Variables	n	n	%	n	%	n	%	n	%		
**Age (years)**											<.0001
18-35	1661	719	7.9	451	12.8	227	8.6	264	12.5		
36-50	4063	1863	20.5	1021	29.0	549	20.7	630	29.9		
51-65	6506	3410	37.5	1334	37.9	997	37.6	765	36.4		
> 65	5134	3098	34.1	713	20.3	878	33.1	445	21.2		
**Educational level**											<.0001
< High-school degree	2479	1407	15.5	386	11.0	493	18.6	193	9.2		
High-school degree	2268	1214	13.4	429	12.2	402	15.2	223	10.6		
Undergraduate degree	5746	2982	32.8	1210	34.4	890	33.6	664	31.6		
Graduate degree	6701	3402	37.4	1458	41.4	840	31.7	1001	47.6		
Unknown	170	85	0.9	36	1.0	26	1.0	23	1.1		
**Monthly income (€ per household)**											<.0001
< 1430	1059	546	6.0	206	5.8	188	7.1	119	5.7		
1430 to 2700	4047	2252	24.8	738	21.0	625	23.6	432	20.5		
2700 to 4800	6255	3245	35.7	1331	37.8	923	34.8	756	35.9		
>= 4800	3165	1557	17.1	704	20.0	416	15.7	488	23.2		
Unknown	498	233	2.6	115	3.3	78	2.9	72	3.4		
Did not wish to answer	2340	1257	13.8	425	12.1	421	15.9	237	11.3		
**Reside with partner during the lockdown**											<.0001
Yes	11908	6060	66.7	2464	70.0	1949	73.5	1435	68.2		
No	1431	709	7.8	349	9.9	189	7.1	184	8.7		
No partner	4025	2321	25.5	706	20.1	513	19.3	485	23.1		
**Children or grandchildren at home during the lockdown [yes]**	3744	1767	19.4	900	25.6	537	20.3	540	25.7		<.0001
**Professional activity changes during lockdown**											<.0001
No professional activity (unemployed, retired, homemaker, maternity leave) or no professional activity during the last seven days (short-term leave).	10764	5907	65.0	1984	56.4	1712	64.6	1161	55.2		
No change	1807	907	10.0	307	8.7	421	15.9	172	8.2		
Yes, change (working from home, new job)	4410	2094	23.0	1147	32.6	447	16.9	722	34.3		
Other	383	182	2.0	81	2.3	71	2.7	49	2.3		
**Essential providers or workers during lockdown**											<.0001
Yes	2565	1289	14.2	482	13.7	485	18.3	309	14.7		
No	14040	7418	81.7	2861	81.3	2083	78.6	1678	79.8		
Don't know	759	383	4.2	176	5.0	83	3.1	117	5.6		
**Residential area during the lockdown (number of inhabitants)**											<.0001

Urban area > 100,000	3449	1931	21.2	637	18.1	363	13.7	518	24.6	
Urban area >= 20,000 to 100,000	3969	2246	24.7	750	21.3	470	17.8	503	23.9	
Urban area < 20,000	4011	2151	23.7	783	22.2	589	22.2	488	23.2	
Rural area	5935	2762	30.4	1349	38.3	1229	46.4	595	28.3	
**Smoking status**										0.0049
Never smoker	7990	4184	46.0	1594	45.3	1187	44.8	1025	48.7	
Former smoker	8135	4260	46.9	1658	47.1	1254	47.3	963	45.8	
Current smoker	1198	626	6.9	254	7.2	208	7.8	110	5.2	
Unknown	41	20	0.2	13	0.4	2	0.1	6	0.3	
**Current weight status**										0.0010
Overweight	5424	2912	32.0	1011	28.8	881	33.2	620	29.5	
Non-overweight	11840	6128	67.4	2483	70.6	1758	66.3	1471	69.9	
Unknown	100	50	0.6	25	0.7	12	0.4	13	0.6	
**Chronic disease [yes]**	6152	3380	37.2	1094	31.1	959	36.2	719	34.2	<.0001
**PHQ-9 (depressive symptoms)**										<.0001
Mild	3872	2115	23.3	728	20.7	451	17.0	578	27.5	
Minimal	11884	6032	66.4	2541	72.2	2016	76.0	1295	61.5	
Moderate	1064	601	6.6	182	5.2	128	4.8	153	7.3	
Moderately severe to severe	544	342	3.8	68	1.9	56	2.1	78	3.7	
**GAD-7 (anxiety disorders)**										<.0001
Mild	3502	1909	21.0	681	19.4	444	16.7	468	22.2	
Minimal	12383	6341	69.8	2602	73.9	2006	75.7	1434	68.2	
Moderate	949	525	5.8	171	4.9	126	4.8	127	6.0	
Severe	530	315	3.5	65	1.8	75	2.8	75	3.6	

Table S2. Characteristics of the men participants according to changes in physical activity during the lockdown

	Men (n = 6194)									
	**Total**	**DECREASE**		**INCREASE**		**OTHERS**		**STABLE**		**P Value**
		3336	*(53.9)*	*983*	*(15.9)*	*480*	*(7.7)*	*1395*	*(22.5)*	
**Variables**	**n**	**n**	**%**	**n**	**%**	**n**	**%**	**n**	**%**	
**Age (years)**										<.0001
18-35	274	131	3.9	55	5.6	49	10.2	39	2.8	
36-50	921	475	14.2	193	19.6	127	26.4	126	9.0	
51-65	1849	994	29.8	334	34.0	146	30.4	375	26.9	
> 65	3150	1736	52.0	401	40.8	158	32.9	855	61.3	
**Educational level**										<.0001
< High-school degree	1346	702	21.0	176	17.9	46	9.6	422	30.2	
High-school degree	684	377	11.3	101	10.3	60	12.5	146	10.5	
Undergraduate degree	1608	871	26.1	259	26.3	125	26.0	353	25.3	
Graduate degree	2504	1357	40.7	441	44.9	245	51.0	461	33.0	
Unknown	52	29	0.9	6	0.6	4	0.8	13	0.9	
**Monthly income (€ per household)**										<.0001
< 1430	211	114	3.4	29	3.0	14	2.9	54	3.9	
1430 to 2700	1173	621	18.6	165	16.8	81	16.9	306	21.9	
2700 to 4800	2745	1504	45.1	396	40.3	204	42.5	641	45.9	
>= 4800	1471	770	23.1	311	31.6	141	29.4	249	17.8	
Unknown	73	73	2.2	17	1.7	4	0.8	17	1.2	
Did not wish to answer	521	521	15.6	65	6.6	36	7.5	128	9.2	
**Reside with partner during the lockdown**										0.0028
Yes	4882	2608	78.2	799	81.3	365	76.0	1110	79.6	
No	238	136	4.1	43	4.4	26	5.4	33	2.4	
No partner	1074	592	17.7	141	14.3	89	18.5	252	18.1	
**Children or grandchildren at home during the lockdown [yes]**	917	471	14.1	194	19.7	104	21.7	148	10.6	<.0001
**Professional activity changes during lockdown**										<.0001
No professional activity (unemployed, retired, homemaker, maternity leave) or no professional activity during the last seven days (short-term leave).	4422	2406	72.1	642	65.3	275	57.3	1099	78.8	
No change	442	221	6.6	60	6.1	41	8.5	120	8.6	
Yes, change (working from home, new job)	1262	679	20.4	266	27.0	156	32.5	161	11.5	
Other	68	30	0.9	15	1.5	8	1.7	15	1.1	
**Essential providers or workers during lockdown**										<.0001
Yes	517	258	7.7	87	8.9	49	10.2	123	8.8	
No	5498	2980	89.3	866	88.1	417	86.9	1235	88.5	
Don't know	179	98	2.9	30	3.0	14	2.9	37	2.7	
**Residential area during the lockdown (number of inhabitants)**										<.0001
Urban area > 100,000	1176	1176	35.2	178	18.1	118	24.6	157	11.3	
Urban area >= 20,000 to 100,000	1330	1330	39.9	174	17.7	119	24.8	228	16.4	

Urban area < 20,000	1527	1527	45.8	252	25.6	121	25.2	323	23.1	
Rural area	2161	973	29.2	379	38.6	122	25.4	687	49.2	
**Smoking status**										0.0087
Never smoker	2127	1137	34.1	345	35.1	202	42.1	443	31.8	
Former smoker	3669	1998	59.9	569	57.9	255	53.1	847	60.7	
Current smoker	389	196	5.9	67	6.8	23	4.8	103	7.4	
Unknown	9	5	0.1	2	0.2	0	0	2	0.1	
**Current weight status**										0.0804
Overweight	2806	1498	44.9	456	46.4	193	40.2	659	47.2	
Non-overweight	3359	1827	54.8	521	53.0	283	59.0	728	52.2	
Unknown	29	11	0.3	6	0.6	4	0.8	8	0.6	
**Chronic disease [yes]**	2779	1517	45.4	432	43.9	171	35.625	659	47.2	0.0016
**PHQ-9 (depressive symptoms)**										<.0001
Mild	774	442	13.2	115	11.7	87	18.125	130	9.3	
Minimal	5179	2747	82.3	835	84.9	362	75.4	1235	88.5	
Moderate	156	93	2.8	24	2.4	23	4.8	16	1.1	
Moderately severe to severe	85	54	1.6	9	0.9	8	1.7	14	1.0	
**GAD-7 (anxiety disorders)**										<.0001
Mild	697	442	13.2	104	10.6	68	14.2	110	7.9	
Minimal	5284	2747	82.3	846	86.1	390	81.3	1248	89.5	
Moderate	141	93	2.8	28	2.8	15	3.1	23	1.6	
Severe	72	46	1.4	5	0.5	7	1.4	14	1.0	
